# Differentiation between MAMP Triggered Defenses in *Arabidopsis thaliana*

**DOI:** 10.1371/journal.pgen.1006068

**Published:** 2016-06-23

**Authors:** Madlen Vetter, Talia L. Karasov, Joy Bergelson

**Affiliations:** 1 Department of Ecology and Evolution, University of Chicago, Chicago, Illinois, United States of America; 2 Committee on Genetics, Genomics and Systems Biology, University of Chicago, Chicago, Illinois, United States of America; John Innes Centre, UNITED KINGDOM

## Abstract

A first line of defense against pathogen attack for both plants and animals involves the detection of microbe-associated molecular patterns (MAMPs), followed by the induction of a complex immune response. Plants, like animals, encode several receptors that recognize different MAMPs. While these receptors are thought to function largely redundantly, the physiological responses to different MAMPs can differ in detail. Responses to MAMP exposure evolve quantitatively in natural populations of *Arabidopsis thaliana*, perhaps in response to environment specific differences in microbial threat. Here, we sought to determine the extent to which the detection of two canonical MAMPs were evolving redundantly or distinctly within natural populations. Our results reveal negligible correlation in plant growth responses between the bacterial MAMPs EF-Tu and flagellin. Further investigation of the genetic bases of differences in seedling growth inhibition and validation of 11 candidate genes reveal substantial differences in the genetic loci that underlie variation in response to these two MAMPs. Our results indicate that natural variation in MAMP recognition is largely MAMP-specific, indicating an ability to differentially tailor responses to EF-Tu and flagellin in *A. thaliana* populations.

## Author Summary

Specialized receptors encoded by plants detect different components of bacterial machinery, and initiate an immune response. These recognition events are thought to induce largely redundant defense signaling, the magnitude of which varies quantitatively among populations, perhaps in response to environment specific differences in microbial threat. Here, we sought to determine whether plants evolve distinct or shared responses to two canonical MAMPs within natural populations. We comprehensively tested the extent of functional redundancy in the response of 186 genotypes of *Arabidopsis thaliana* to variants of each of two classes of bacterial signals, flagellin and EF-Tu. Although plants respond similarly to recognition of different variants of the same MAMP, we found the response to one MAMP class to be largely uncorrelated with the response to the other class. We further investigated the genetic bases underlying growth changes to determine whether similar genes contribute to variation in the response to EF-Tu and flagellin bacterial signals. We find limited genetic similarity, revealing novel MAMP-specific signaling components. The differentiation of these responses reveals MAMP-specific fine tuning of the immune response.

## Introduction

Pathogens pose a constant threat to their hosts. While lacking the adaptive immune system present in mammals, plants have evolved a two-tiered immune system of considerable specificity. The first tier of defense involves the recognition of microbe associated molecular patterns (MAMPs) that are common to many microbes. Plants recognize MAMPs, such as the elongation factor Tu (EF-Tu) and flagellin, or their epitopes elf18 and flg22, by specialized receptors that allow the plant to discriminate self versus non-self and induce signaling cascades that result in defense responses [1, 2].

The second tier of the plant immune system involves the recognition of specific microbial strains (in contrast to semi-universal microbial patterns) via the activity of resistance proteins. The specificity of the immune system in distinguishing specific microbes was previously thought to lie primarily in the activity of resistance proteins. Recent studies have challenged this paradigm in demonstrating both qualitative and quantitative variation in MAMP perception [3].

Plant hosts have the capacity to recognize multiple MAMPs, which helps ensure that at least one of the many MAMPs is recognized. For example, the order Brassicales evolved the capacity to recognize the MAMP elf18 in addition to ancient flg22, that is detected by all land plants [4–6]. The perception systems for elf18 and flg22 share common molecular components, such as the co-receptor BAK1 [7, 8], and elicit similar changes in gene expression [2, 9] suggesting that MAMP-triggered signal transduction and the associated signaling cascade converge quickly, regardless of the MAMP trigger.

If MAMP-perception systems were completely redundant one would expect identical physiological responses to distinct MAMP triggers. However, some physiological responses differ in detail. For example, elf18 and flg22 induce different Ca2+ signaling and macroscopic growth responses [10]. In particular, flg22 perception induces an equally strong growth reduction in roots and shoots, whereas elf18 perception acts more strongly in leaves than in roots [10, 11]. Additional evidence hints at a more complex interplay of molecular components after induction with different MAMPs [12–15].

The differentiated responses to separate MAMP classes suggest that the benefit of recognizing multiple classes may extend beyond redundancy. For example, it is possible that plants tailor defense response intensities to different MAMPs, an adaptation that could be especially beneficial when MAMP composition reflects differences in microbial community composition. Our previous work revealed extensive natural variation in the detection of a variant of flg22 and signatures of selection at the genetic loci underlying this variation [3]. This quantitative variation in MAMP perception indicated that these traits are evolving within natural populations. Whether these traits–the perception and response to divergent MAMPs–act and evolve redundantly is an open question.

To determine whether detection of flagellin and EF-Tu evolve redundantly in natural populations, we tested a large panel of *A. thaliana* genotypes for their response to different variants of these two MAMPs. As a read-out for MAMP perception, we utilized the macroscopic phenotype of seedling growth inhibition (SGI), a phenotypic response shown to correlate closely with MAMP perception in several *Brassicaceae* species [3]. In assaying quantitative variation in the responses to different MAMP variants and different MAMP classes, and determining the genetic loci underlying this variation via genome-wide association mapping, we shed light on the evolution of this first tier of defense.

## Results and Discussion

### Natural variation in MAMP-induced seedling growth inhibition

**Fig 1:**
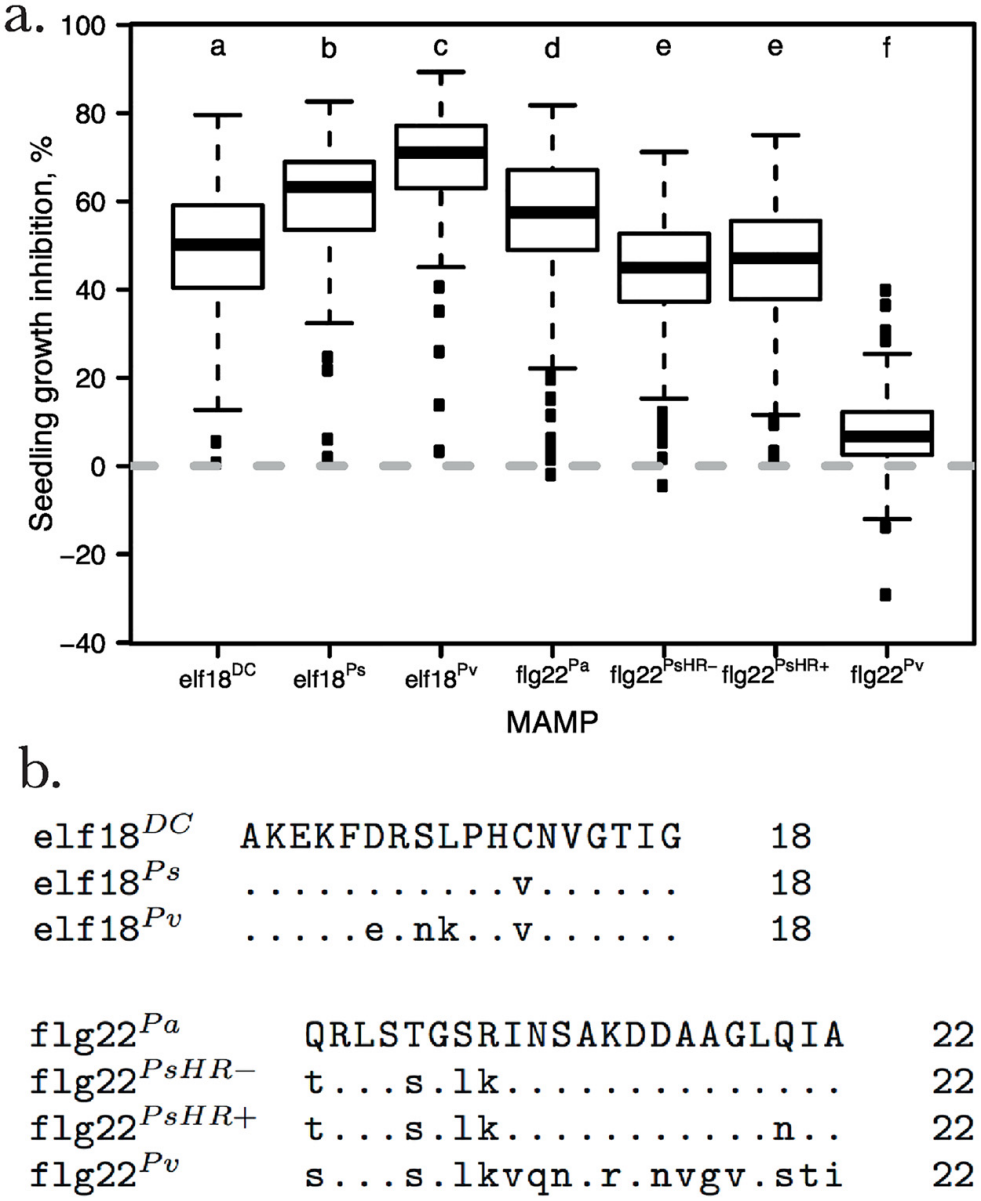
MAMP recognition varies quantitatively between MAMP alleles and between *A. thaliana* genotypes. (a) Three elf18-variants and four flg22 variants were used to trigger SGI in 186 genotypes of *A. thaliana*. The x-axis indicates the MAMP variant and bacteria of origin: *Pseudomonas syringae* pv. *tomato* DC3000 (DC), *P. syringae* (PsHR-, PsHR+), *P. viridiflava* (Pv), and *P. aeruginosa* (Pa). Plotted are mean SGI values of 186 genotypes. The mean SGI for each *A. thaliana* genotype is estimated by calculating the relative reduction of fresh mass in percent. At least three replicates were measured per genotype and treatment to calculate mean SGI. The horizontal bar of the boxplot represents the median, the edges of the box present the 25th and 75th percentile. The whiskers are drawn at the data point that is closest to 1.5 x interquartile range. All outliers are shown. Small letters above the boxes indicate statistically different groups (ANOVA/Tukey’s post hoc test). (b) The peptide sequences of both elf18 and flg22 differ between closely related Pseudomonads (*P. syringae* and *P. viridiflava*) and within *P. syringae*.

In order to characterize natural variation in MAMP-induced seedling growth inhibition (SGI) in *A. thaliana*, we challenged 186 natural genotypes with several peptide variants for each of two classes of MAMPs, elf18 and flg22 (Fig 1). An important feature of the study system is that growth inhibition is triggered by the peptides alone, without any confounding effects of bacterial proliferation and disease.

We observed extensive variation in MAMP-induced SGI: Some genotypes exhibited no response while others exhibited a mean reduction in growth of up to 86% (e.g., Dra3-1 in response to elf18^*Pv*^). Different MAMP peptides induced significantly different SGI (ANOVA *F*_5,5828_ = 854, *p* = < 2*e* − 16, see S1 Table for full ANOVA table), consistent with previous findings [16–18].

In particular, the highly diverged peptide flg22^*Pv*^ (Fig 1b) induced little to no SGI in most genotypes (Fig 1a). Although this flg22 variant of *P. viridiflava* induced no response in most *A. thaliana* genotypes, its elf18 variant induced the strongest SGI of all elf18 variants. In this case, the independent response to *P. viridiflava* enables the continued detection of pathogenic invader even though recognition of one MAMP fails.

**Fig 2:**
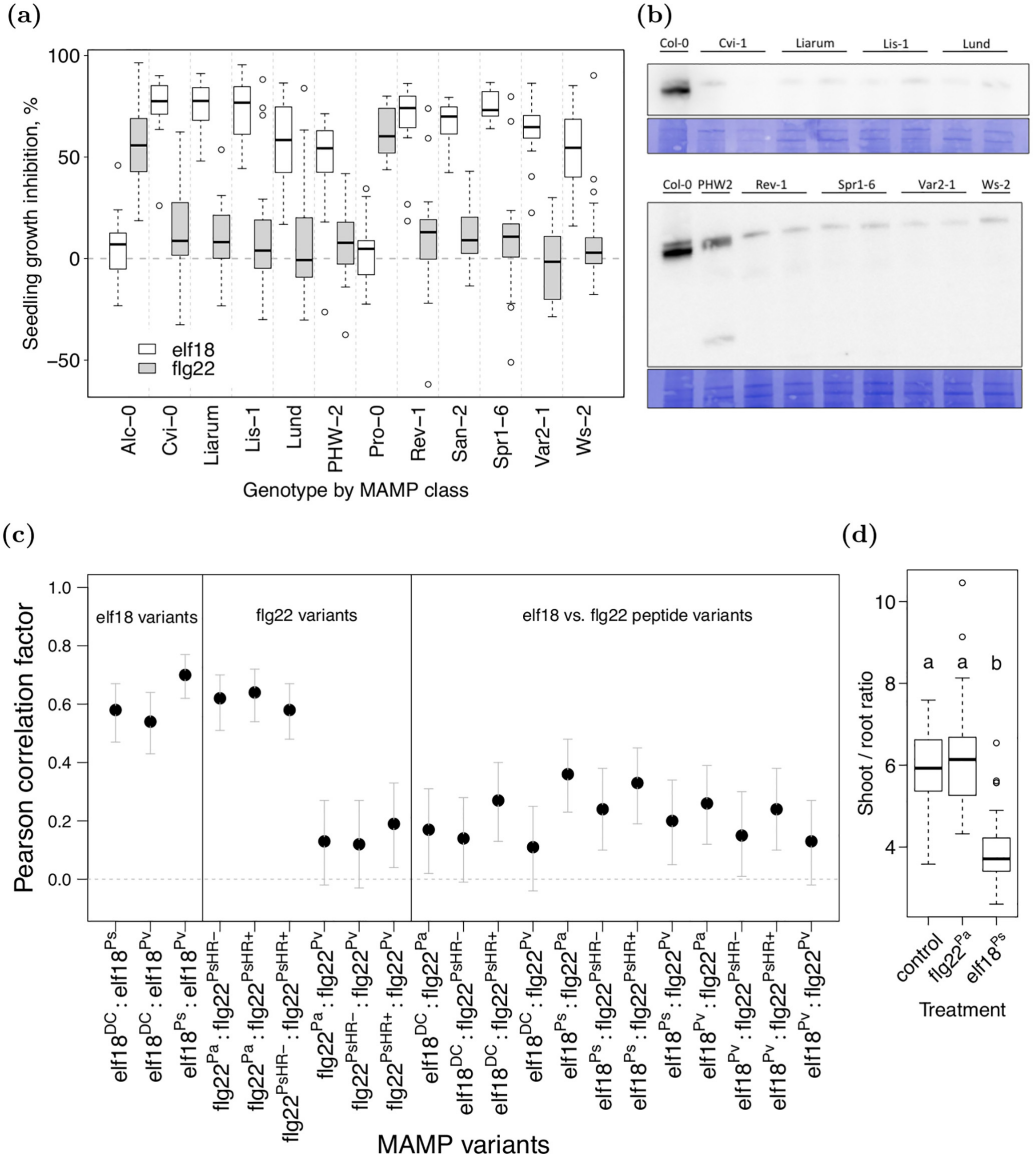
Different genetic basis of variation in the response to elf18 vs. flg22. (a) Genotypes that showed no SGI to one MAMP class are not impaired in their growth response to the other MAMP class. We classified genotypes as flg22-insensitive when they exhibited less than 15% SGI over all peptides in one MAMP class. The boxplots present SGI induced by the two MAMP classes (i.e., elf18^*DC*^, elf18^*Ps*^ and elf18^*Ps*^ grouped as“elf18” and flg22^*Pa*^, flg22^*PsHR*+^ and flg22^*PsHR*−^ grouped as “flg22”). (b) Genotypes with low flg22-induced SGI exhibit reduced FLS2 protein abundance or a truncated FLS2 protein (PHW2). Two separate immunoblots and their corresponding coomassie colloidal blue stained membranes for total protein loading control. Immunoblots were conducted with an anti-FLS2 antibody that is directed against the kinase region of the flg22 receptor FLS2. We sequenced this region to ensure that the epitope recognized by the anti-FLS2 antibody is identical to the Col-0 control and thus allows direct comparison of protein levels. Note that the genotype San-2 also exhibited less than mean 15% SGI in response to the flg22 MAMP variants. (c) Pearson correlation coefficients and confidence intervals for genotype means of SGI. S2 Table contains the corresponding numerical values. (d) Effect of different MAMPs on plant architecture. Plants (N > 40) were grown in presence or absence of 100nM of MAMP and both fresh mass and shoot / root ratio determined. ANOVA, followed by a post hoc Tukey test, revealed significantly different means (*p* ≤ 0.05; indicated by different letters above the box plots).

Two of the 186 *A. thaliana* genotypes did not exhibit elf18-induced SGI and 10 did not exhibit flg22-induced SGI (Fig 2b). Genotypes lacking a response to one MAMP class were not impaired in their response to the other class, suggesting that loss-of-function of the elf18 and flg22 perception systems undergo differentiated evolution. In order to investigate the cause of low flg22-induced SGI, we tested FLS2 protein levels of nine genotypes that showed little or no SGI in response to any flg22 peptide variant. We observed compromised protein production in all tested genotypes (Fig 2d). It remains unclear if lower protein levels are caused by mutations within the protein coding sequence of the receptor or trans-acting factors. Analysis of mRNA expression levels in a subset of the flg22-insensitive genotypes revealed lower levels of mRNA expression of *FLS2* (S1 Fig) in these genotypes. We furthermore obtained *FLS2* nucleotide sequences for 9 of the 10 flg22-insensitive genotypes. Six out of nine show putative deletions in the catalytic site of the serine-threonine kinase domain of *FLS2* (S1 Text). These results suggest that the absence of SGI in response to flg22 treatment is largely explained by differences in the coding sequence and the abundance of the receptor itself. The comparatively high prevalence of flg22-insensitive genotypes raise the possibility that the elf18 perception system has surpassed the ancient flg22 pathway [4] in importance, at least in *A. thaliana*.

Finally, it is interesting to note that small differences in amino acid sequence of the inducing peptides had the potential to profoundly impact SGI. For example, three amino acid differences between elf18^*Ps*^ and elf18^*Pv*^ resulted in an 18% increase in mean SGI (Welch’s t-test *t* = −7.0, *p* = 1.24*e*^−11^). On the other hand, flg22^*PsHR*−^ and flg22^*PsHR*+^, which differ by a single amino acid, did not cause statistically significant differences in SGI (Welch’s t-test *t* = −0.89, *p* = 0.37, Fig 1a).

### Correlation of SGI in response to different MAMP variants

We asked if genotypes exhibit similar SGI in response to variants of flg22 and elf18. If these MAMPs act redundantly, one would expect a strong correlation among the induced physiological responses. We observed strong correlations among variants within each MAMP class: the average Pearson correlation coefficient within a MAMP class is 0.61 whereas the mean correlation of SGI between MAMP classes is much weaker (mean *R* = 0.24, see Fig 2a and S2 Table for significance and confidence intervals of individual comparisons).

Veluchamy et. al [19] observed a similar disparity between within-class and between-class correlations in a small set of heirloom tomato tested for reactive oxygen production upon treatment with three MAMPs, flg22^*Pa*^, flgII-28 and csp22. Overall, the weak correlation between classes of MAMP peptides suggests some autonomy in the underlying genetic bases.

### Genetic basis of MAMP-induced SGI

**Fig 3:**
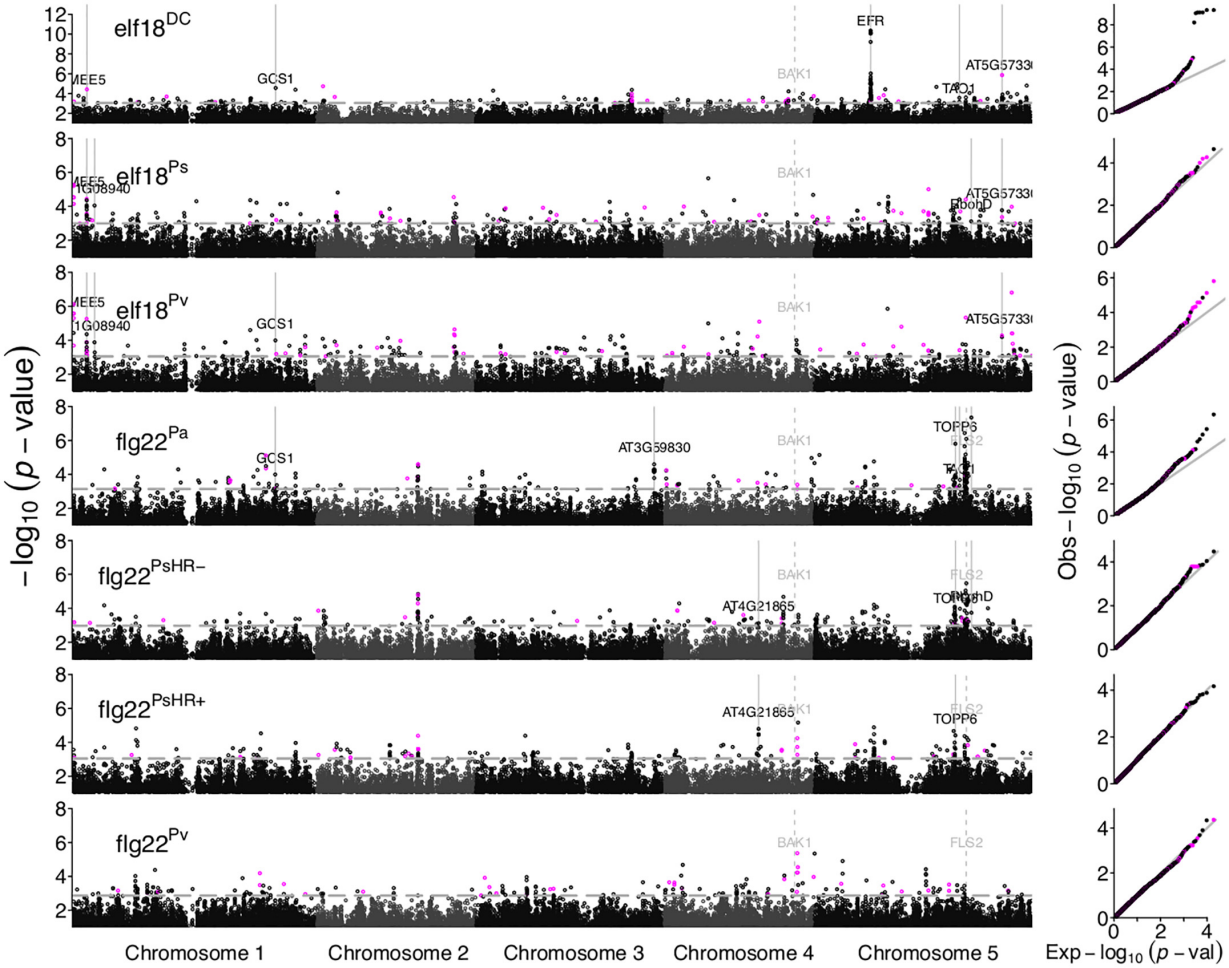
Manhattan plots of the GWA mapping for SGI induced by seven MAMP variants. The genome-wide distribution of the -log10 *p* -values of the SNP—phenotype associations are plotted as a function of the genomic position along the five chromosomes. The GWA study was conducted using EMMAX, which controls for population structure. The *x*-axis displays the position along the chromosome while the *y*-axis displays the *p*-value from a linear mixed model regression associating a given SNP with SGI. SNPs with minor allele frequency (MAF) < 0.05 were excluded. For clarity, only SNPs characterized by -log10(*p*-value) ≤ 1 are shown. The horizontal dashed line depicts the threshold for the 0.1% tail of the *p*-value distribution, which varies between peptide variants (flg22^*Pv*^: 0.00134 to elf18^*DC*^: 0.00088). SNPs that displayed above-threshold *p*-values were considered for further analysis. SNPs corresponding to rare alleles (0.05 > MAF > 0.1) are plotted in magenta. The right-most panel shows quantile plots of the expected versus observed *p*-values for each MAMP variant. Note the different scale of the y-axis of the uppermost panel. Genes that could be validated as underlying the natural variation in SGI are indicated in black. The *a priori* candidate genes *FLS2* and *BAK1* are not significantly associated with SGI but are indicated in gray for reference. A table with all genes underlying the different peak regions is available in the data folder of the repository bitbucket.org/mvetter/geneticbasissgi.

We performed genome-wide association mapping to a) reveal the genetic basis underlying natural variation in SGI and b) identify loci that are uniquely important in shaping either flg22- or elf18-induced SGI (Fig 3). We first calculated marker-based heritability estimates (*h*^2^ = 0.29 on average for all perceived peptides, S3 Table) to test if SGI is amenable to GWA mapping [20]. The MAMP flg22^*Pv*^ induced little to no SGI in most *A. thaliana* genotypes, which is reflected in a broad-sense heritability estimate of 0 (S3 Table).

We tested the significance of associations between the seven phenotypes and approximately 210,000 genome-wide SNP markers [21] in linear mixed models (EMMAX) that takes population structure into account [22]. We identified a number of loci that exhibit significant association with natural variation in SGI (Fig 3). In general, SGI appears to be governed by a complex genetic architecture involving loci with small effect. Only the GWA mapping of elf18^*DC*^-induced SGI identified a locus of large putative effect; this locus, which was found on chromosome 5, explains 28% of the observed variation.

**Fig 4:**
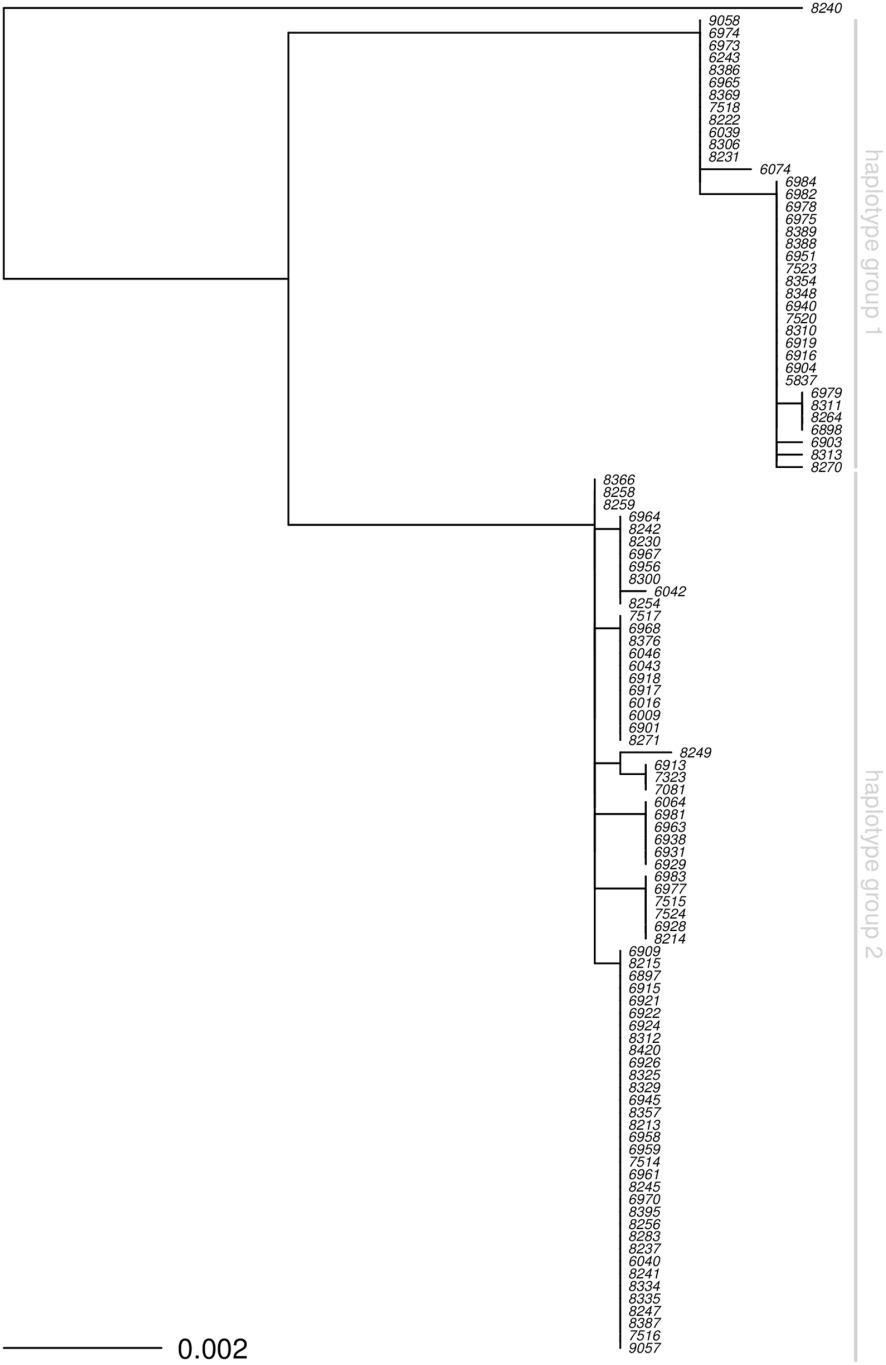
The maximum-likelihood phylogram of EFR protein coding sequence reveals two distinct haplotype groups. Phylogenetic analysis of 3,096 nucleotides of the *EFR* coding region of 109 *A. thaliana* genotypes that were also represented in the GWA mapping. The sequences were reconstructed from SNP data of the 1001 genome project (http://1001genomes.org/). All nodes are supported by 100 out of 100 bootstrap replicates. Vertical grey bars indicate two haplotype groups that exhibit 28 nucleotide differences. Genotype 8240 (i.e., Kulturen-1) is a strong outlier with 54 nucleotide differences compared to haplotype group 2.

The MAMP receptors *EFR* and *FLS2* were considered strong *a priori* candidate genes for variation in SGI. The top SNPs of the single prominent peak for elf18^*DC*^-induced SGI, indeed, co-localize with the *EFR* locus. Surprisingly, *EFR* is not strongly associated with variation in SGI induced by the other two elf18 variants, elf18^*Ps*^ and elf18^*Pv*^. To investigate this further, we conducted phylogenetic analysis and revealed two major *EFR* haplotype groups (Fig 4) that strongly differentiate detection of elf18^*DC*^ from elf18^*Ps*^ and elf18^*Pv*^ (Figs 3 and S2). Thus, EFR controls natural variation in SGI for only a subset of elf18 variants.

Variation in flg22-induced SGI was attributed to many loci with small effect. The most prominent peak (*p* ≤ 4 * 10^−8^) in the flg22^*Pa*^-induced SGI co-localizes with the *a priori* candidate gene NADPH/respiratory burst oxidase protein D (*RbohD*), that is known to fine tune reactive oxygen production and hypersensitive response around pathogen infection sites. None of the SNPs within the 30 kb window comprising the *FLS2* gene are significantly associated with flg22-induced SGI (see close-up of genetic region in S3 Fig.) Similarly, no significantly associated SNP mapped near the FLS2 co-receptor BAK1 (Fig 3). The functional importance of BAK1 for flg22-induced immunity is well-established [7]; however, the unusually limited natural genetic variation at the *BAK1* locus (*π* = 0.0008 in 80 genotypes published in [23] versus 0.005 for a genome wide estimate by [24]) does not appear to contribute to natural variation in the SGI.

We identified a number of *a priori* candidate genes that are known to have an effect on SGI (Table 1). These candidates were found to be enriched in the 0.1% tail of elf18^*DC*^ and elf18^*Ps*^ but not the other MAMPs (Table 2). Thus, our results illustrate that genes with strong phenotypic effects in knock-out experiments do not necessarily harbor genetic variation causing natural variation in this plant phenotype [25].

**Table 1:** *A priori* candidate genes that statistically associate with natural variation in seedling growth inhibition. Candidate genes were identified within 15 kb around a SNP falling in the 0.1% tail of EMMAX *p*-values. The distance of the candidate locus and the highest associate SNP are indicated in bp (column distance).

Gene name	AGI	GWA p-value	MAF	nb SNPs	MAMP	distance
ATRABA1B	AT1G16920	0.000393	0.45	1	flg22^*Pa*^	7845
AtRABA6a	AT1G73640	0.000591	0.24	1	flg22^*PsHR*+^	6987
HBI1	AT2G18300	0.000840	0.16	1	elf18^*DC*^	-14709
HBI1	AT2G18300	0.000232	0.07	2	elf18^*Ps*^	-11445
ATMKK5	AT3G21220	0.000876	0.18	1	flg22^*PsHR*+^	0
ERD2B	AT3G25040	0.000574	0.09	1	elf18^*Ps*^	-32
RIN4	AT3G25070	0.000574	0.09	1	elf18^*Ps*^	-8038
RIN4	AT3G25070	0.000277	0.17	1	elf18^*Pv*^	5701
MPK3	AT3G45640	0.000057	0.18	1	elf18^*Pv*^	-1043
AT3G55450	AT3G55450	0.000567	0.10	1	elf18^*Ps*^	6010
ATGSL05	AT4G03550	0.000388	0.39	1	elf18^*DC*^	-1785
MAPKKK9	AT4G08480	0.000626	0.25	1	elf18^*DC*^	-1693
MAPKKK9	AT4G08480	0.000002	0.25	2	elf18^*Ps*^	-1693
MAPKKK9	AT4G08480	0.000010	0.25	1	elf18^*Pv*^	-1693
ARA7	AT4G19640	0.000773	0.07	1	elf18^*Ps*^	8533
CIB1	AT4G34530	0.000007	0.26	1	flg22^*PsHR*+^	-1861
CIB1	AT4G34530	0.000028	0.06	2	flg22^*Pv*^	-3035
BSK1	AT4G35230	0.000788	0.28	1	elf18^*Pv*^	-12160
BRI1	AT4G39400	0.000408	0.06	1	elf18^*Ps*^	7898
EFR	AT5G20480	0.000000	0.34	43	elf18^*DC*^	-785
RbohD	AT5G47910	0.000768	0.18	1	elf18^*Ps*^	2845
RbohD	AT5G47910	0.000000	0.18	1	flg22^*Pa*^	2845
RbohD	AT5G47910	0.000181	0.18	1	flg22^*PsHR*−^	2845
RSW3	AT5G63840	0.000805	0.19	1	elf18^*DC*^	3893

**Table 2:** The traits elf18^*DC*^- and elf18^*Ps*^-induced SGI map a significant number of *a priori* candidate genes. We assembled a list of 77 genes that have published evidence to alter SGI in response to either elf18 or flg22. The measure ‘frequency’ indicates how many *a priori* candidate genes were found among the number of GWA candidate genes (nb. genes) for all peaks of each trait. The empirical *p*-value is generated by shifting the number of peaks along the genetic positions and calculating the frequency of found *a priori* genes in the number of genes under these shifted peaks. The random shift along the genetic position maintains patterns of linkage disequilibrium and was repeated 100 times.

MAMP	nb. peaks	nb. genes	a priori	frequency	emp. p-value
elf18^*DC*^	110	922	5	0.0054	0.04
elf18^*Ps*^	127	1103	8	0.0073	0.01
elf18^*Pv*^	130	1084	4	0.0037	0.14
flg22^*Pa*^	96	845	2	0.0024	0.48
flg22^*PsHR*−^	108	923	1	0.0011	0.86
flg22^*PsHR*+^	104	907	3	0.0033	0.28
flg22^*Pv*^	126	1071	1	0.0009	0.91

### MAMP variants induce distinct physiological responses

We examined the genetic overlap in peaks identified during GWAS analysis of the responses to each MAMP. While MAMPs within each class share a higher number of peaks than expected by chance (S4 Table and S4 Fig), we did not observe enrichment in the overlap of GWAS peaks for SGI induced by the MAMP classes elf18 and flg22. This finding is consistent with our observation of high correlations of SGI within, but not between, MAMP classes, and suggests a common genetic architecture within each of two distinct MAMP classes.

Our results raise the possibility that flg22 and elf18 induce molecular responses that are more differentiated than is suggested by their similar patterns of gene expression [2, 9]. The observation that elf18 and flg22 induce different macroscopic changes further supports the differentiation of these responses. Specifically, flg22 acts both on leaf and root tissue, while elf18 is most effective on leaves [10, 11]. We confirmed this observation in a quantitative experiment demonstrating that application of elf18 significantly alters the shoot/root ratio compared to untreated plants, whereas flg22 treatment does not (ANOVA *F*_2,175_ = 77.0, Tukey post hoc elf18^*Ps*^ vs control *p* ≤ 2.2*e*^−16^, flg22^*Pa*^ vs control *p* = 0.36, Fig 2c).

### MAMP-induced SGI is fine tuned by both shared and distinct loci

To further test our hypothesis that different loci underlie natural variation in elf18 and flg22-induced SGI, we selected candidate genes from our GWA for experimental validation using both MAMP classes. For each MAMP, we selected the 0.1% tail of *p*-values (i.e., the 203 SNPs most strongly associated with MAMP-induces SGI). To account for linkage disequilibrium, we defined peak regions that comprise all SNPs within 15 kb to either side of the SNP with the lowest *p*-value. This resulted on average in 113 peak regions per MAMP. On average, six genes underlie a peak region, resulting in 4481 genes across all peak regions and six MAMPs (flg22^*Pv*^ excluded, see S5 Table for details). For experimental confirmation of candidates, we focused on genes that were either mapped in multiple MAMPs, genes with differential expression upon MAMP treatment [2, 9] or with a known role in defense mechanism or growth. We retrieved one T-DNA insertion line for each of 88 genes from the ABRC seed stock center and successfully confirmed homozygosity of T-DNA insertion for 57 mutant lines that were tested for SGI. We identified 11 candidate genes that significantly alter SGI (Table 3), some of which are *a priori* candidates such as *EFR* and *RbohD*, while others are genes of unknown function (e.g., AT4G21865 and AT5G57345). We consider as confirmed only candidates that pass *q* ≤ 0.05 after correction for false discovery rate. This approach reduces the reporting of false positives but might exclude some true positives (e.g, RIN4, involved in modulating flg22-induced SGI [26].) Our confirmation of 11 genes reveals a four fold enrichment in significant loci in comparison to the null. Our confirmation rate of 20% (after correction for multiple testing) is therefore substantially higher than expected by chance.

**Table 3:** The majority of confirmed loci exhibit MAMP-specific responses. The first column indicates the candidate gene name. The columns elf18 and flg22 denote whether differential SGI was observed in response to either MAMP in mutant plants (an X indicates that a member of the peptide class induced differential SGI significance at FDR < 0.05). The column “Eff” denotes if SGI was stronger (+) or weaker (-) in the mutant in comparison to the wild-type Col-0. The magnitude of response differs between several of the validated candidates (see S6 Table). Information from the Arabidopsis Information Resource (www.arabidopsis.org) is given for genes that have not been previously associated with SGI.

Gene name	elf18	flg22	Eff	Function
MEE5	X		+	MATERNAL EFFECT EMBRYO ARREST 5 encodes a protein with similarity to splicing factor Snu114 with putative translation factor activity and function in RNA binding.
AT1G08940	X		+	Phosphoglycerate mutase family protein that putatively regulates flavonoid biosynthesis, protein targeting to membrane and regulation of plant-type hypersensitive response.
GCS1	X	X	+/-	Glucosidase I is a component of the oligosaccharide processing pathway in the endoplasmatic reticulum. It catalyzes the first step of oligosaccaride processing, a post-translational modification which adds oligosaccharide chains to proteins. This modification is crucial for proper function of MAMP receptors [27–29]. GCS1 might affect post-translational modification of the MAMP receptors EFR and FLS2 or indispensable co-factors.
AT3G59830		X	+	Integrin-linked protein kinase family, with serine/threonine/tyrosine kinase activity, putatively involved in integrin-mediated signaling pathway, plant-type cell wall modification, pollen tube development, pollen tube growth, protein phosphorylation and regulation of signal transduction.
AT4G21865	X	X	+	Protein with unknown function that is putatively expressed in guard cells.
EFR	X		-	EF-Tu receptor [2].
TOPP6		X	+	TYPE ONE SERINE/THREONINE PROTEIN PHOSPHATASE 6, putatively involved in protein dephosphorylation in the chloroplast.
TAO1	X	X	-	Target of AvrB operation 1 is an *R*-gene that contributes to disease resistance induced by the *Pseudomonas syringae* effector AvrB.
RbohD	X	X	-	NADPH oxidase plays a role in the production of reactive oxygen species and signaling processes upon detection of several MAMPs [30].
AT5G57330		X	-	Galactose mutarotase-like superfamily protein that is putatively involved in glucosinolate biosynthetic process and response to abscisic acid.
AT5G57345	X		+	Protein of unknown function, putatively involved in cellular cation homeostasis through divalent metal ion transport.

Of the 11 confirmed candidates, four impact both elf18- and flg22-induced SGI and seven control either elf18- or flg22-induced SGI (Table 3). This finding suggests differences in the molecular pathways involved in the recognition of and response to different MAMP classes. The discrepancy between the extensive overlap previously identified in the pathways for elf18 and flg22 recognition, and the reduced overlap in genes identified here, may be explicable by either of two hypotheses.

One possibility is that loci underlying natural variation in SGI are primarily involved in the initial steps of MAMP perception prior to signal convergence. Functional differences in the receptor alone might be caused by sequence variation in its protein coding region [3], its transcriptional regulation [15], its post-translational glycolysation [27, 28] or modification of obligatory co-receptors [31]. Another possibility is that yet unidentified pathways, unique to perception of one MAMP, lead to observed phenotypic differences. We found some support for this hypothesis by identifying a number of experimentally confirmed loci that alter SGI uniquely upon treatment with elf18 variants but not flg22 variants.

Overall, our results reveal a lack of correlation between a plant’s response to elf18 and flg22. What are the implications of this uncorrelated response? Natural populations experience fluctuations in both abiotic and biotic conditions, and plants in different environments are colonized by disparate microbial communities and pathogen species. In light of this variability, the decoupling of elf18- and flg22-triggered physiological responses is potentially advantageous. Genotypes that respond more strongly to one MAMP do not in general respond equally strongly to another MAMP i.e., there are no genotypes hypersensitive to all MAMPs. This uncoupling of the responses allows for responses to different MAMPs to evolve independently in populations. There is also the recent demonstration of epistatic effects of recognizing multiple MAMPs, at least in mammalian systems [32]. The ways in which hosts regulate their microbiomes is a complex issue that is only beginning to be understood, but distinctly tailored MAMP initiated defense is likely one contributing factor.

## Materials and Methods

All data and custom scripts are available at https://bitbucket.org/mvetter/geneticbasissgi/

### Identification of natural MAMP sequences

Plants perceive specific epitopes of EF-Tu and flagellin that are known as elf18 and flg22, respectively. Flg22 sequences of five natural *P. viridiflava* strains [33] were identified using primers 5’-GCCATCGCGACGATAACTA-3’ and 5’-GGCGTTTTCGTTGATGTTCT-3’. Flg22 sequences for *P. viridiflava* strains LP23.1a and RMX3.1b as well as both elf18 and flg22 sequences for 20 *P. syringae* strains isolated from *A. thaliana* and the surrounding plant *Drava verna* [34] were derived from genome sequences available in the Bergelson laboratory (S2–S3 Texts). The *Pseudomonas* strains from which these MAMPs were derived were previously isolated from natural populations of *A. thaliana*. Infection analyses with the *P. syringae* strains determined that a subset of strains did not induce HR on *A. thaliana* [34, 35]. We tested MAMP variants derived from strains that did not induce HR (HR-) and strains that successfully induce HR (HR+).

The identified elf18 and flg22 variants were synthesized by EZBiolab, Carmel, IN.

### Plant culture and estimation of SGI

Seedlings growth inhibition (SGI) was estimated for 186 genotypes of *A. thaliana* that were part of the panel in Atwell et al. [21]. Plants grown in the absence (control) or presence of 100nM MAMP (treatment) were grown in pairs and cultivated in sterile conditions as described in Vetter et al. [3]. Seedling growth inhibition was calculated as relative reduction of fresh mass in percent by [(CFM—TFM) / CFM] * 100, where CFM stands for control fresh mass and TFM for treatment fresh mass. Phenotypic values of SGI were calculated by averaging at least three pairs of control and MAMP treatments per genotype; these pairs were obtained in five independent biological trials for elf18 and seven independent biological trials for flg22 (resulting in a maximum number of 15 or 21 biologically independent values of SGI per genotype).

All data analysis was conducted in R [36]. Particular packages are indicated where appropriate.

### SDS-PAGE and immunoblotting

Plant material was grown and processed as described in Vetter et al. [3]. In short, nitrogen frozen plant material was homogenized, weighed and dissolved in equal amounts of extraction buffer (25mM MES pH 6, 3mM MgCl_2_, 10mM NaCl and Sigma protease inhibitor, 2% SDS). Proteins were separated in a Novex NuPage 3–8% Tris-acetate gel and blotted onto a PVDF membrane (Immobilon, Millipore). The α-FLS2 antibody was incubated in 1:5,000 dilution, overnight at 4°. A horseradish peroxidase, coupled to secondary anti-rabbit IgG in 1:2,000 dilution was used to detect FLS2 protein bound to the membrane. This antibody was previously demonstrated to be specific to FLS2 [37].

### mRNA and nucleotide sequence analysis of *FLS2*

Expression data for the *FLS2* locus were obtained from [38] and the correlation between SGI and mRNA expression level were determined for those genotypes for which we had both SGI and RNA-sequencing information (49 accessions). The *FLS2*nucleotide sequence data for the genotypes that failed to respond to flg22 was obtained from http://tools.1001genomes.org/pseudogenomes/.

### Correlation of SGI in response to diverse MAMPs

We investigated SGI induced by three elf18 and four flg22 peptide variants in 186 genotypes of *A. thaliana*. Twelve genotypes exhibited a mean SGI < 15% in response to either elf18 or flg22 variants. We considered these genotypes natural SGI mutants and excluded them prior to correlation analysis. We calculated Pearson correlation coefficients and confidence intervals for the remaining 174 genotypes. Note that no genotype failed to recognize both MAMPs.

### Estimates of heritability

Marker-based heritability and confidence intervals were calculated using the marker_h2 function of the package ‘heritability’ in R [39]. This model incorporates a genetic relatedness matrix and generates REML-estimates of the additive genetic variance ($\sigma_{A}^{2}$), residual variance ($\sigma_{E}^{2}$) and their standard errors, which allows calculation of heritability according to the equation, $h^{2}=\sigma_{A}^{2}/\left(\sigma_{A}^{2}+\sigma_{E}^{2}\right)$. The relatedness matrix was generated using plink1.07 [40, 41] and SNP data by Kim et al. [42].

### Genome-wide association mapping

Genome-wide association mapping was conducted on average SGI for each genotype for which high density genotype data was available [42]. Analysis and restructuring of genotype data was conducted in plink1.07 [40, 41]. The mixed linear model EMMAX takes population structure into account by incorporating a K matrix of genetic relatedness [22]. EMMAX positively biases association signals for alleles with low frequency in the population. We therefore disregarded SNPs with a minor allele frequency (MAF) smaller than 5%. Our mapping panel contained 214,051 discriminative SNPs, of which 203,498 passed the MAF filter.

### Investigation of the genetic architecture

In order to assess genetic similarities within and between MAMP classes, we identified shared peak regions. To account for linkage disequilibrium [42] and the fact that the highest associated SNP might not be the causal one [43], we defined peak regions encompassin 15 kb on either side of the highest associated SNP. A literature search identified 77 *a priori* candidate genes with published evidence of their participation in the SGI response to either elf18 or flg22. We tested for enrichment of these *a priori* candidates among our identified peak regions by calculating their frequency among the genes underlying the mapped peak regions of each trait. In order to assign an empirical *p*-value, we kept the number of peaks and their genetic distance intact but slid them across the genetic position of each chromosome. We then counted the number of genes underlying these shifted peaks and calculated the frequency of *a priori* candidates within the shifted peak regions. We repeated this procedure 100 times and determined the number of times we achieved an equal or greater frequency of *a priori* candidate genes. All custom scripts are available at https://bitbucket.org/mvetter/geneticbasissgi/.

### Selection and confirmation of candidate genes

We considered SNPs that fell in the 0.1% tail of ranked GWA *p*-values; these correspond to the 203 most strongly associated SNPs. To account for linkage disequilibrium, we defined peak regions 15 kb to either side of the highest associated SNP and identified genes co-localizing with these peak regions based on TAIR 9 annotation. From this list of 4481 genes, we selected candidate genes for experimental confirmation if they fulfilled at least one of the following criteria: (1) detected by multiple MAMP variants, (2) differentially expressed upon MAMP perception (using data of [2, 9]), or (3) *a priori* candidate for growth or defense related processes. We selected 88 loci for which T-DNA insertion mutants were ordered from ABRC seed stock center (list available in the data folder of the repository bitbucket.org/mvetter/geneticbasissgi.) We analyzed a single T-DNA insertion line for each candidate gene (but see [44]) and could confirm homozygosity of T-DNA insertion for 57 mutant lines using primers according to http://signal.salk.edu/tdnaprimers.2.html. Silke Robatzek, The Sainsbury Laboratory, Norwich, kindly provided fls2-24 and efr-0 mutants for control purposes. We grew each SALK mutant genotype in a minimum of 12 replicates with an equal number of wild-type (WT) Col-0 or (WT) Ler plants (according to respective mutant background). Significant differences in SGI between mutants and WT were determined using a non-parametric Wilcoxon rank-sum test in combination with multiple testing correction using the fdrtool package [45]. To identify MAMP-specific SGI, we first tested the MAMP class and variant that led in the GWA to the candidate gene selection. If a significant result was observed, we tested at least one more variant within the same class as well as a variant in the other class.

## Supporting Information

S1 Fig The relationship between receptor expression level and SGI. Expression data on the x-axis for *FLS2* (a) and *EFR* (b) are taken from [38]. Plants were grown at 16°C. Receptor mRNA expression level is plotted against SGI induced by the respective MAMP. Expression data is presented in the units of reads per kilobase per million of mapped reads (RPKM). The red dotted line denotes the cutoff for distinguishing MAMP-sensitive from MAMP-insensitive genotypes (15% SGI.) Genotypes that did not respond to flg22 treatment exhibit low expression of *FLS2*.
(PDF)

S2 Fig The *EFR* haplotype group strongly influences elf18^*DC*^-induced SGI. The three panels shows SGI induced by elf18^*DC*^, elf18^*Ps*^ and elf18^*Pv*^ in 186 genotypes of *A. thaliana*. Above each panel we indicate results of a t-test testing the effect of *EFR* haplotype group on SGI. We labeled the two outliers (i.e., elf18-insensitive genotypes) Pro-0 and Alc-0 in the plot. The red dot highlights mean SGI of the genotype Col-0. While elf18^*DC*^-induced SGI is strongly determined by the *EFR* haplotype group, elf18^*Ps*^ and elf18^*Pv*^ are influenced to a lesser extant. This leads to a strong genotype-phenotype association (i.e., peak) in our GWA for elf18^*DC*^ but not elf18^*Ps*^ or elf18^*Pv*^ at the *EFR* locus.
(PDF)

S3 Fig Genomic region 18500000 to 19600000 of chromosome 5 is densely populated with highly associated SNPs. The genomic region 18500000 to 19600000 of chromosome 5 has several peaks that are associated with flg22-induced SGI. These peaks do not co-localize with known *a priori* candidate genes such as flagellin receptor *FLS2*. None of the 69 SNPs within 15 kb to either side of *FLS2* is significantly associated with flg22-induced SGI. The genes associated with individual peaks are stored in the data folder of the repository bitbucket.org/mvetter/geneticbasissgi.
(PDF)

S4 Fig The number of shared peaks within a MAMP class is higher than expected by chance. Each histogram shows the distribution of the number of shared peaks for 100 GWA runs that were generated with randomized phenotypic values. The red vertical line represents the number of peaks that were found in the actual mapping. Given is also the empirical *p*-value that indicates the chance of identifying the observed number of shared peaks by chance. *P*-values ≤ 0.002 are considered significant after Bonferroni correction for multiple testing.
(PDF)

S1 Table Analysis of variance determining the effect of MAMP class (MAMPclass), host genotype (genotype) and MAMP peptide (MAMP, nested within MAMP class) on seedling growth inhibition (SGI). (PDF)

S2 Table Correlation of SGI is high within MAMP classes (elf18 or flg22) but not among MAMP classes. The table indicates Pearson’s correlation coefficients for genotype means of seedling growth inhibition. Genotypes that did not exhibit seedling growth inhibitionin response to elf18 or flg22 were excluded prior analysis. Significant correlations are indicated in bold (Bonferroni corrected p < 0.006).
(PDF)

S3 Table Heritability estimates and confidence intervals. Marker-assisted heritability was estimated for seedling growth inhibition (SGI), fresh mass in control conditions (CFM) and fresh mass after MAMP treatment (TFM). SGI is calculated by [(CFM—TFM) / CFM] * 100.
(PDF)

S4 Table Number of shared genomic regions. Many genomic regions were mapped by more than one MAMP variant. SGI induced by peptides of the same MAMP class (elf18 or flg22 variants) share a larger number of genomic regions than between MAMP classes. As a result of linkage disequilibrium and SNP density, a highly associated SNP can be located several kb away from the causal gene. We therefore considered genomic regions of 30 kb instead of directly comparing shared genes.
(PDF)

S5 Table Summary statistics of GWA mapping. We conducted GWA mapping on SGI, induced by seven diverse MAMPs. We considered the 0.1% tail of strongest associated p-values for further analysis, which corresponds to 203 SNPs out of 203,498 for which genotype data were available. Column “MAMP” indicates the MAMP used to induce SGI, “*p*-value” indicates the *p*-value cut-off (i.e., the *p*-value of the 203rd strongest associated SNP), “nb. peaks” indicates to how many peaks these 203 SNPs cluster. The column “nb. of genes” indicates how many genes were contained in these peak regions. Peak regions are defined as genomic areas 15 kb to either side of the highest associated SNP.
(PDF)

S6 Table Experimental validation of GWA candidate loci. We tested plants carrying a non-functional allele (mutant) versus plants carrying the WT allele (Col-0 except fls2-24 that has a L*er* genetic background). Column N indicates the number of tested mutants / WT plants, and effect (Eff) indicates an increase (+) or decrease (-) in SGI. Column ‘*p*-value’ indicates statistically discernible difference of seedling growth inhibition in mutant from WT using the non-parametric Wilcoxon rank-sum test. In order to correct for multiple testing, *p*-values derived by the Wilcoxon rank-sum test were re-evaluated by false discovery rate (FDR). *P*-values or FDR-corrected values smaller than 0.05 are printed in bold. The *FLS2* locus was not associated with MAMP-induced seedling growth inhibition but the fls2-24 mutant was included for control purposes.
(PDF)

S1 Text flg22-insensitivity is associated with nucleotide deletions within the *FLS2* Serine-Threonine kinase catalytic domain. Shown are the 100 bp surrounding the start site of the Serine/Threonine kinase catalytic domain of *FLS2* in twenty two genotypes that exhibit SGI in response to flg22 (SGI+) and nine genotypes that do not (SGI-). Pseudogenome data was extracted from http://tools.1001genomes.org/pseudogenomes/. Brown shading details the beginning of the kinase domain. Eight of the nine SGI- genotypes contain missing data in the first 50 bp of this domain (N’s) and six contain stretches of N’s greater than 2 bp, suggestive of deletions in these genotypes.
(XLS)

S2 Text Sequences of Flagellin in *P. syringae* and *P. viridiflava*. Fasta nucleotide sequences of region surrounding flagellin gene in 20 *P. syringae* strains and two *P. viridiflava* strains. Strains are labeled with name first then species designation.
(TXT)

S3 Text Sequences of EF-Tu in *P. syringae* and *P. viridiflava*. Fasta nucleotide sequences of region surrounding EF-Tu gene in 20 *P. syringae* strains and two *P. viridiflava* strains. Strains are labeled with name first then species designation.
(TXT)
