# Supplementary figures and images for: Differentiation between MAMP Triggered Defenses in Arabidopsis thaliana
Source: PLoS Genet. 2016 Jun 23;12(6):e1006068. doi: 10.1371/journal.pgen.1006068 (PMC4919071; doi:10.1371/journal.pgen.1006068)

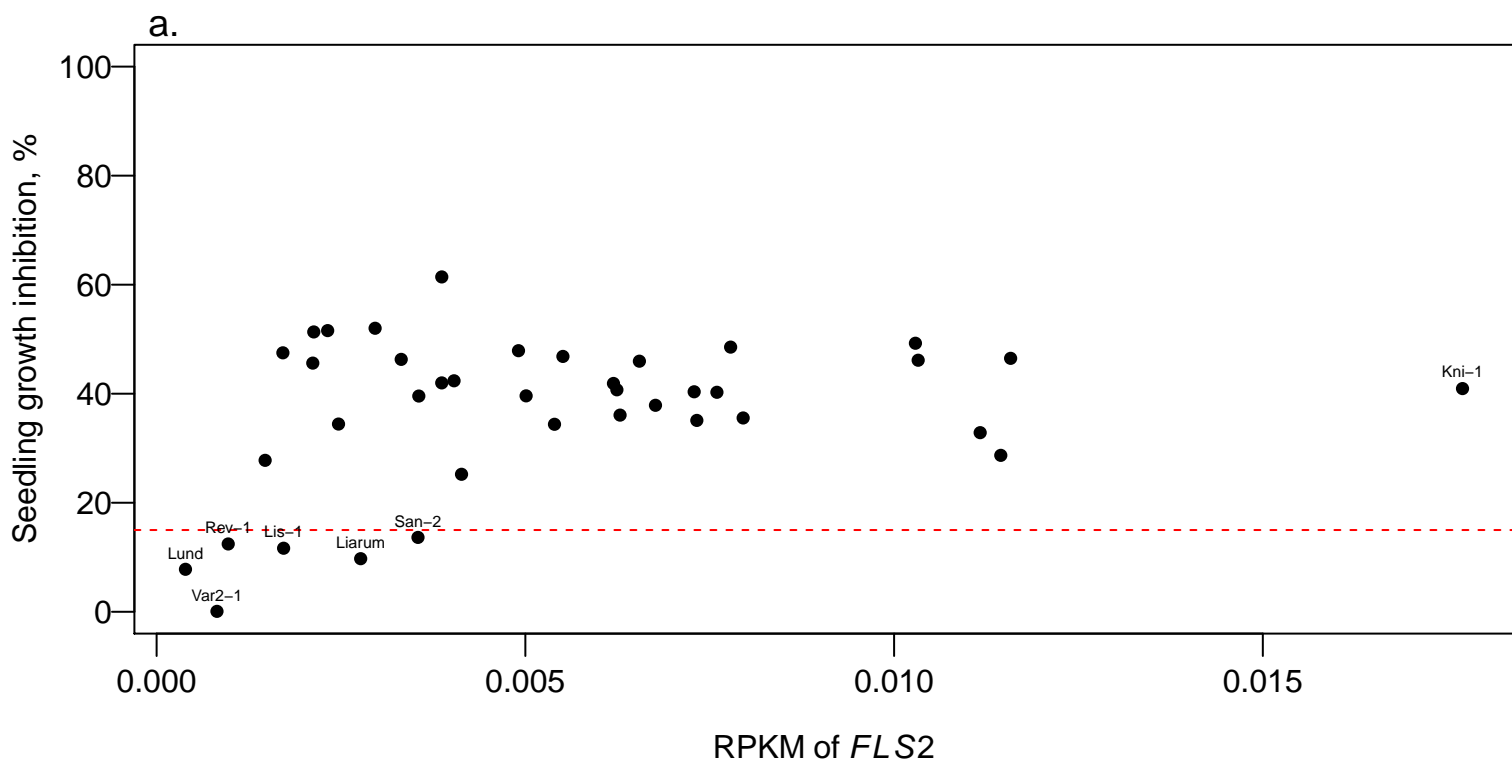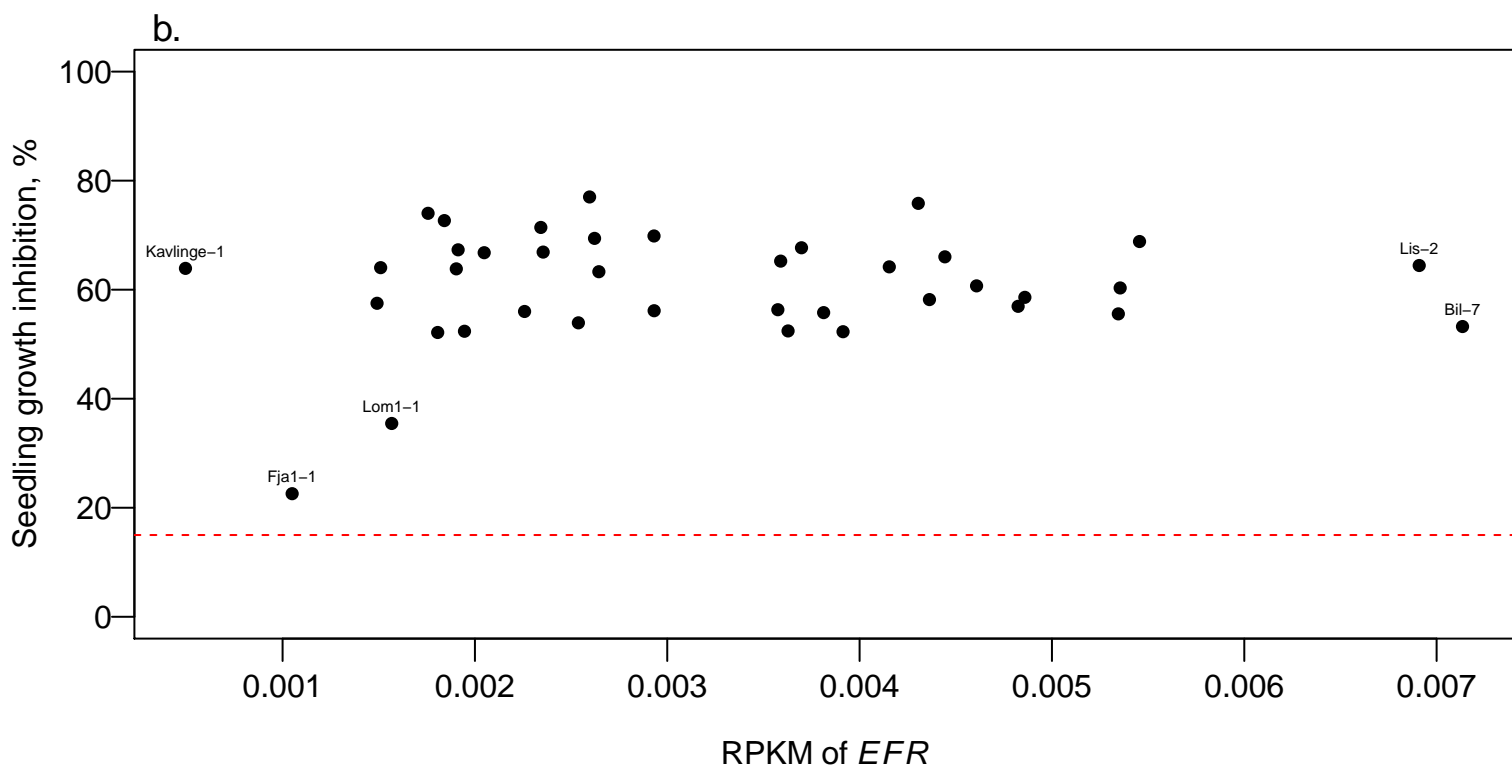

Supplement: S1 Fig — Expression data on the x-axis for FLS2 (a) and EFR (b) are taken from [38]. Plants were grown at 16°C. Receptor mRNA expression level is plotted against SGI induced by the respective MAMP. Expression data is presented in the units of reads per kilobase per million of mapped reads (RPKM). The red dotted line denotes the cutoff for distinguishing MAMP-sensitive from MAMP-insensitive genotypes (15% SGI.) Genotypes that did not respond to flg22 treatment exhibit low expression of FLS2. (PDF) [file pgen.1006068.s001.pdf]

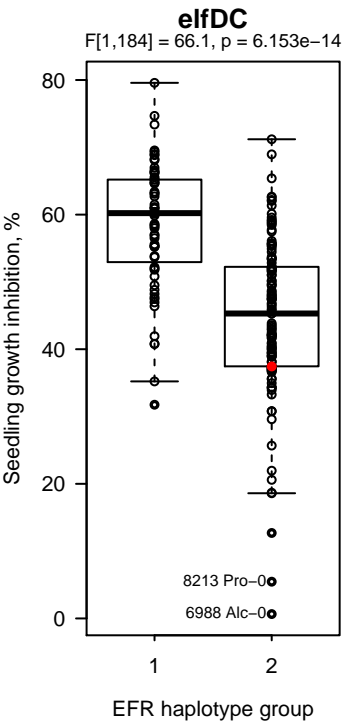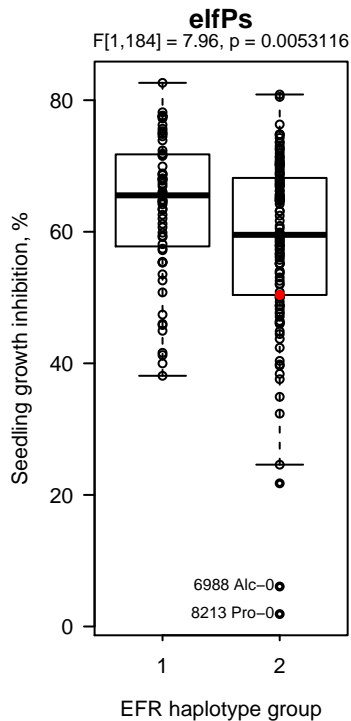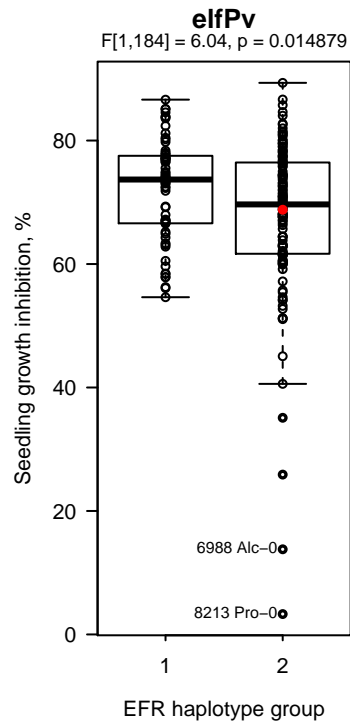

Supplement: S2 Fig — The three panels shows SGI induced by elf18DC, elf18Ps and elf18Pv in 186 genotypes of A. thaliana. Above each panel we indicate results of a t-test testing the effect of EFR haplotype group on SGI. We labeled the two outliers (i.e., elf18-insensitive genotypes) Pro-0 and Alc-0 in the plot. The red dot highlights mean SGI of the genotype Col-0. While elf18DC-induced SGI is strongly determined by the EFR haplotype group, elf18Ps and elf18Pv are influenced to a lesser extant. This leads to a strong genotype-phenotype association (i.e., peak) in our GWA for elf18DC but not elf18Ps or elf18Pv at the EFR locus. (PDF) [file pgen.1006068.s002.pdf]

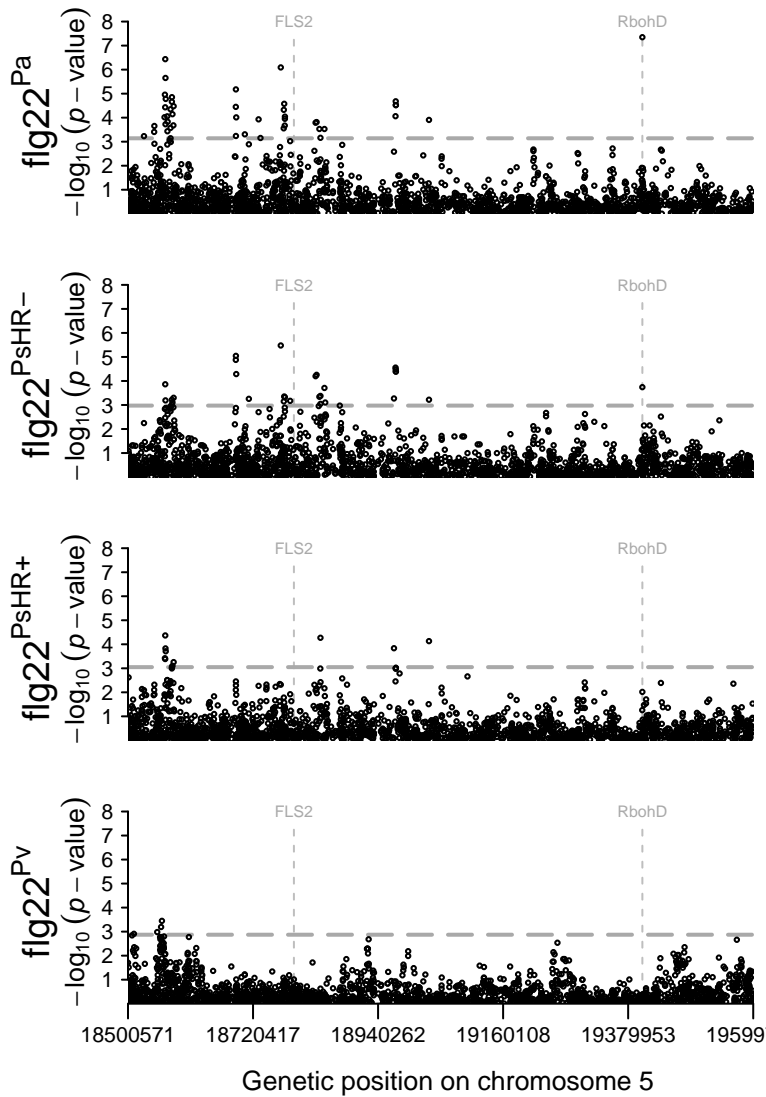

Supplement: S3 Fig — The genomic region 18500000 to 19600000 of chromosome 5 has several peaks that are associated with flg22-induced SGI. These peaks do not co-localize with known a priori candidate genes such as flagellin receptor FLS2. None of the 69 SNPs within 15 kb to either side of FLS2 is significantly associated with flg22-induced SGI. The genes associated with individual peaks are stored in the data folder of the repository bitbucket.org/mvetter/geneticbasissgi. (PDF) [file pgen.1006068.s003.pdf]
