# Supplementary material for: Differentiation between MAMP Triggered Defenses in Arabidopsis thaliana
Source: PLoS Genet. 2016 Jun 23;12(6):e1006068. doi: 10.1371/journal.pgen.1006068 (PMC4919071; doi:10.1371/journal.pgen.1006068)
Supplement: S4 Fig — Each histogram shows the distribution of the number of shared peaks for 100 GWA runs that were generated with randomized phenotypic values. The red vertical line represents the number of peaks that were found in the actual mapping. Given is also the empirical p-value that indicates the chance of identifying the observed number of shared peaks by chance. P-values ≤ 0.002 are considered significant after Bonferroni correction for multiple testing. (PDF) [file pgen.1006068.s004.pdf]

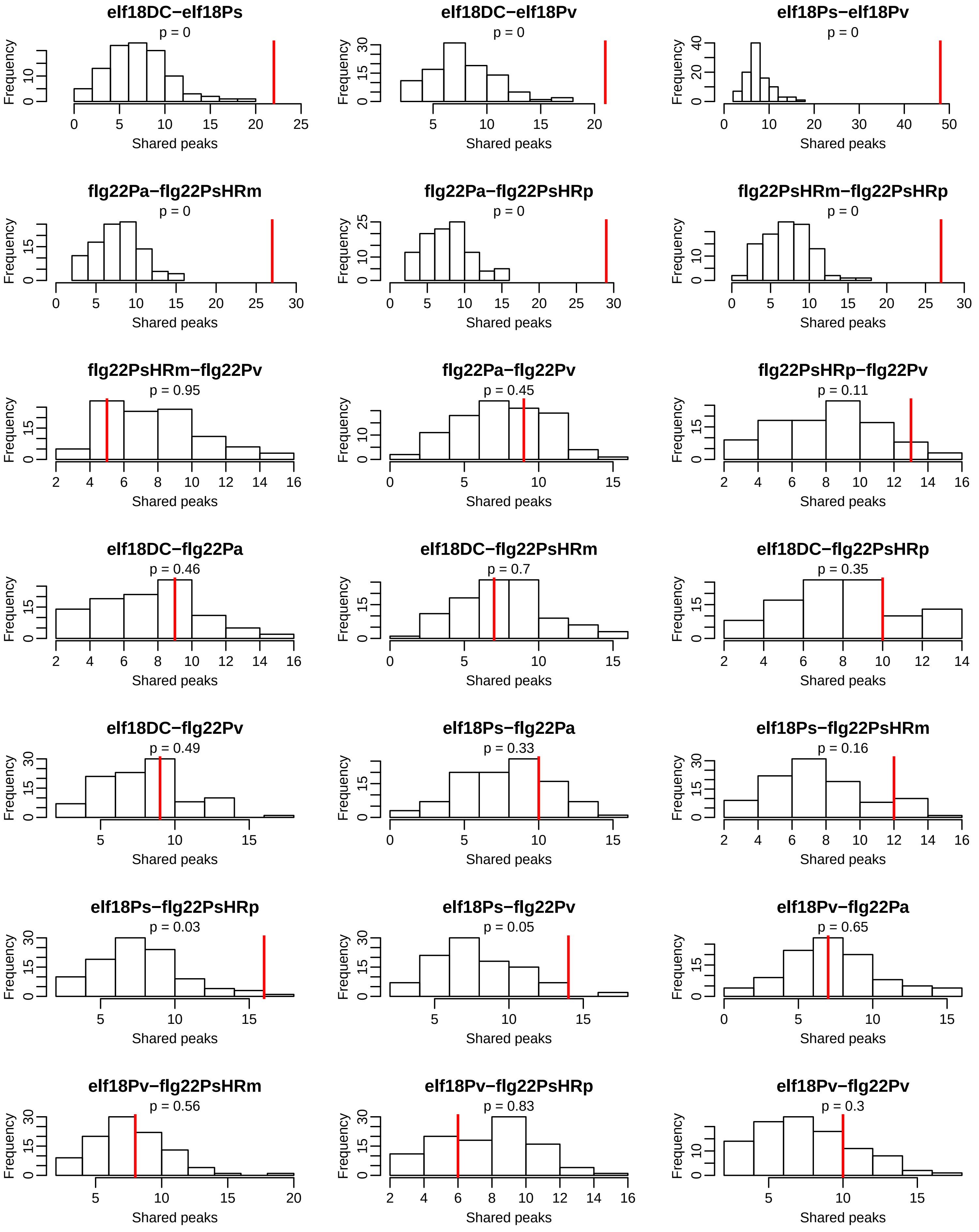
elf18DC−elf18Ps
p = 0
elf18DC−elf18Pv
p = 0
elf18Ps−elf18Pv
p = 0
flg22Pa−flg22PsHRm
p = 0
flg22Pa−flg22PsHRp
p = 0
flg22PsHRm−flg22PsHRp
p = 0
flg22PsHRm−flg22Pv
p = 0.95
flg22Pa−flg22Pv
p = 0.45
flg22PsHRp−flg22Pv
p = 0.11
elf18DC−flg22Pa
p = 0.46
elf18DC−flg22PsHRm
p = 0.7
elf18DC−flg22PsHRp
p = 0.35
elf18DC−flg22Pv
p = 0.49
elf18Ps−flg22Pa
p = 0.33
elf18Ps−flg22PsHRm
p = 0.16
elf18Ps−flg22PsHRp
p = 0.03
elf18Ps−flg22Pv
p = 0.05
elf18Pv−flg22Pa
p = 0.65
elf18Pv−flg22PsHRm
p = 0.56
elf18Pv−flg22PsHRp
p = 0.83
elf18Pv−flg22Pv
p = 0.3
Frequency
Shared peaks
