# Supplementary material for: Differentiation between MAMP Triggered Defenses in Arabidopsis thaliana
Source: PLoS Genet. 2016 Jun 23;12(6):e1006068. doi: 10.1371/journal.pgen.1006068 (PMC4919071; doi:10.1371/journal.pgen.1006068)
Supplement: S1 Table — (PDF) [file pgen.1006068.s005.pdf]

	Df	Sum Sq	Mean Sq	F value	Pr(>F)
MAMPclass	1	743214.93	743214.93	1910.28	0.0000
genotype	185	486672.12	2630.66	6.76	0.0000
MAMPclass:MAMP	5	1661595.01	332319.00	854.16	0.0000
MAMPclass:genotype	185	340286.26	1839.39	4.73	0.0000
MAMPclass:MAMP:genotype	925	419731.46	453.76	1.17	0.0008
Residuals	5828	2267445.86	389.06		
