# Supplementary material for: Differentiation between MAMP Triggered Defenses in Arabidopsis thaliana
Source: PLoS Genet. 2016 Jun 23;12(6):e1006068. doi: 10.1371/journal.pgen.1006068 (PMC4919071; doi:10.1371/journal.pgen.1006068)
Supplement: S2 Table — The table indicates Pearson’s correlation coefficients for genotype means of seedling growth inhibition. Genotypes that did not exhibit seedling growth inhibitionin response to elf18 or flg22 were excluded prior analysis. Significant correlations are indicated in bold (Bonferroni corrected p < 0.006). (PDF) [file pgen.1006068.s006.pdf]

	elf18^{DC}	elf18^{Ps}	elf18^{Pv}	flg22^{Pa}	flg22^{PsHR-}	flg22^{PsHR+}	flg22^{Pv}
elf18^{DC}		**0.58**	**0.54**	0.17	0.14	**0.27**	0.11
elf18^{Ps}	**0.58**		**0.70**	**0.36**	**0.24**	**0.33**	0.20
elf18^{Pv}	**0.54**	**0.70**		**0.26**	0.15	**0.24**	0.13
flg22^{Pa}	0.17	**0.36**	**0.26**		**0.62**	**0.64**	0.13
flg22^{PsHR-}	0.14	**0.24**	0.15	**0.62**		**0.58**	0.12
flg22^{PsHR+}	**0.27**	**0.33**	**0.24**	**0.64**	**0.58**		0.19
flg22^{Pv}	0.11	0.20	0.13	0.13	0.12	0.19	
