# Supplementary material for: Differentiation between MAMP Triggered Defenses in Arabidopsis thaliana
Source: PLoS Genet. 2016 Jun 23;12(6):e1006068. doi: 10.1371/journal.pgen.1006068 (PMC4919071; doi:10.1371/journal.pgen.1006068)
Supplement: S3 Table — Marker-assisted heritability was estimated for seedling growth inhibition (SGI), fresh mass in control conditions (CFM) and fresh mass after MAMP treatment (TFM). SGI is calculated by [(CFM—TFM) / CFM] * 100. (PDF) [file pgen.1006068.s007.pdf]

MAMP	SGI	CFM	TFM
elf18^{DC}	0.31 (0.23, 0.38)	0.32 (0.24, 0.39)	0.32 (0.25, 0.4)
elf18^{Ps}	0.3 (0.22, 0.38)	0.28 (0.21, 0.36)	0.32 (0.24, 0.4)
elf18^{Pv}	0.33 (0.25, 0.41)	0.23 (0.16, 0.31)	0.32 (0.24, 0.4)
flg22^{Pa}	0.31 (0.24, 0.38)	0.18 (0.12, 0.24)	0.33 (0.26, 0.4)
flg22^{PsHR-}	0.23 (0.17, 0.3)	0.21 (0.15, 0.27)	0.26 (0.19, 0.33)
flg22^{PsHR+}	0.24 (0.18, 0.31)	0.19 (0.13, 0.26)	0.3 (0.23, 0.36)
flg22^{Pv}	0.01 (-0.03, 0.04)	0.24 (0.17, 0.3)	0.22 (0.16, 0.29)
