# Supplementary material for: Differentiation between MAMP Triggered Defenses in Arabidopsis thaliana
Source: PLoS Genet. 2016 Jun 23;12(6):e1006068. doi: 10.1371/journal.pgen.1006068 (PMC4919071; doi:10.1371/journal.pgen.1006068)
Supplement: S4 Table — Many genomic regions were mapped by more than one MAMP variant. SGI induced by peptides of the same MAMP class (elf18 or flg22 variants) share a larger number of genomic regions than between MAMP classes. As a result of linkage disequilibrium and SNP density, a highly associated SNP can be located several kb away from the causal gene. We therefore considered genomic regions of 30 kb instead of directly comparing shared genes. (PDF) [file pgen.1006068.s008.pdf]

	$elf18^{DC}$	$elf18^{Ps}$	$elf18^{Pv}$	$flg22^{Pa}$	$flg22^{PsHR-}$	$flg22^{PsHR+}$	$flg22^{Pv}$
$elf18^{DC}$	110	22	21	9	7	10	9
$elf18^{Ps}$		127	48	10	12	16	14
$elf18^{Pv}$			130	7	8	6	10
$flg22^{Pa}$				96	27	29	9
$flg22^{PsHR-}$					108	27	5
$flg22^{PsHR+}$						104	13
$flg22^{Pv}$							126
