# Supplementary material for: Differentiation between MAMP Triggered Defenses in Arabidopsis thaliana
Source: PLoS Genet. 2016 Jun 23;12(6):e1006068. doi: 10.1371/journal.pgen.1006068 (PMC4919071; doi:10.1371/journal.pgen.1006068)
Supplement: S5 Table — We conducted GWA mapping on SGI, induced by seven diverse MAMPs. We considered the 0.1% tail of strongest associated p-values for further analysis, which corresponds to 203 SNPs out of 203,498 for which genotype data were available. Column “MAMP” indicates the MAMP used to induce SGI, “p-value” indicates the p-value cut-off (i.e., the p-value of the 203rd strongest associated SNP), “nb. peaks” indicates to how many peaks these 203 SNPs cluster. The column “nb. of genes” indicates how many genes were contained in these peak regions. Peak regions are defined as genomic areas 15 kb to either side of the highest associated SNP. (PDF) [file pgen.1006068.s009.pdf]

| **MAMP** | **p-value** | **nb. peaks** | **nb. of genes** |
|---|---|---|---|
| $elf18^{DC}$ | 0.00089 | 110 | 922 |
| $elf18^{Ps}$ | 0.0010 | 127 | 1103 |
| $elf18^{Pv}$ | 0.00089 | 130 | 1084 |
| $flg22^{Pa}$ | 0.00072 | 96 | 845 |
| $flg22^{PsHR-}$ | 0.00106 | 108 | 923 |
| $flg22^{PsHR+}$ | 0.00089 | 104 | 907 |
| $flg22^{Pv}$ | 0.00134 | 126 | 1071 |
