# Supplementary material for: Differentiation between MAMP Triggered Defenses in Arabidopsis thaliana
Source: PLoS Genet. 2016 Jun 23;12(6):e1006068. doi: 10.1371/journal.pgen.1006068 (PMC4919071; doi:10.1371/journal.pgen.1006068)
Supplement: S6 Table — We tested plants carrying a non-functional allele (mutant) versus plants carrying the WT allele (Col-0 except fls2-24 that has a Ler genetic background). Column N indicates the number of tested mutants / WT plants, and effect (Eff) indicates an increase (+) or decrease (-) in SGI. Column ‘p-value’ indicates statistically discernible difference of seedling growth inhibition in mutant from WT using the non-parametric Wilcoxon rank-sum test. In order to correct for multiple testing, p-values derived by the Wilcoxon rank-sum test were re-evaluated by false discovery rate (FDR). P-values or FDR-corrected values smaller than 0.05 are printed in bold. The FLS2 locus was not associated with MAMP-induced seedling growth inhibition but the fls2-24 mutant was included for control purposes. (PDF) [file pgen.1006068.s010.pdf]

AGI	Genotype	Distance	MAMP	Eff	N	W	p-value	FDR
AT1G01770	*at1g01770*	1553	elf18^{Ps}	-	15/14	153	**0.0367**	0.10
AT1G01770	*at1g01770*	1553	elf18^{Pv}	-	15/7	68	0.30	0.40
AT1G01770	*at1g01770*	1553	flg22^{Pa}	-	23/22	285	0.48	0.49
AT1G01780	*plim2b*	199	elf18^{Ps}	-	21/41	484	0.43	0.47
AT1G06160	*ora59*	12321	elf18^{DC}	-	16/36	398	**0.0289**	0.09
AT1G06160	*ora59*	12321	elf18^{Ps}	+	18/18	127	0.28	0.39
AT1G06160	*ora59*	12321	elf18^{Pv}	-	30/57	954	0.38	0.44
AT1G06160	*ora59*	12321	flg22^{Pa}	+	18/18	99	**0.0471**	0.12
AT1G06220	*mee5*	-3709	elf18^{DC}	+	19/48	253	**0.0042**	**0.0224**
AT1G06220	*mee5*	-3709	elf18^{Ps}	+	38/58	682	**0.0017**	**0.0118**
AT1G06220	*mee5*	-3709	elf18^{Pv}	+	32/43	669	0.84	0.63
AT1G06220	*mee5*	-3709	flg22^{Pa}	-	22/23	276	0.61	0.55
AT1G08940	*at1g08940*	-5623	elf18^{Ps}	+	42/68	879	**0.0007**	**0.0068**
AT1G08940	*at1g08940*	-5623	flg22^{Pa}	+	26/27	234	**0.0375**	0.11
AT1G15670	*at1g15670*	-5698	flg22^{Pa}	-	20/39	454	0.31	0.40
AT1G51460	*abcg13*	0	elf18^{Ps}	+	15/18	118	0.56	0.53
AT1G51460	*abcg13*	0	elf18^{Pv}	-	33/53	710	0.15	0.27
AT1G57670	*at1g57670*	0	elf18^{Ps}	+	24/24	224	0.19	0.32
AT1G58440	*sqe1*	-13842	elf18^{Pv}	-	49/87	2489	0.11	0.22
AT1G60110	*at1g60110*	0	elf18^{DC}	+	62/74	2036	0.26	0.37
AT1G60110	*at1g60110*	0	elf18^{Pv}	-	64/64	2152	0.62	0.56
AT1G60110	*at1g60110*	0	flg22^{Pa}	+	24/23	219	0.23	0.35
AT1G61215	*brd4*	5596	flg22^{Pa}	-	16/32	181	0.10	0.22
AT1G62090	*lrrpk*	2549	elf18^{Ps}	+	11/12	58	0.65	0.57
AT1G62570	*at1g62570*	-103	elf18^{Pv}	+	16/36	291	0.96	0.66
AT1G64900	*cyp89a2*	2665	flg22^{Pa}	-	40/79	1389	0.28	0.39
AT1G67490	*gcs1*	9794	elf18^{DC}	-	18/18	199	0.25	0.37
AT1G67490	*gcs1*	9794	elf18^{Ps}	+	18/18	119	0.18	0.31
AT1G67490	*gcs1*	9794	elf18^{Pv}	-	39/61	1568	**0.0076**	**0.0346**
AT1G67490	*gcs1*	9794	flg22^{Pa}	+	42/64	840	**0.0012**	**0.0097**
AT1G67490	*gcs1*	9794	flg22^{PsHR-}	+	6/6	0	**0.0022**	**0.0133**
AT1G67510	*at1g67510*	-1072	elf18^{DC}	-	16/36	349	0.23	0.35
AT1G67510	*at1g67510*	-1072	elf18^{Pv}	+	34/70	1107	0.57	0.53

AT1G73805	*sard1*	1880	elf18^{DC}	-	28/47	786	0.16	0.29
AT1G73805	*sard1*	1880	elf18^{Pv}	-	19/41	431	0.52	0.51
AT2G01450	*mpk17*	2364	flg22^{PsHR-}	+	32/56	803	0.42	0.46
AT2G03250	*at2g03250*	12867	elf18^{Ps}	-	22/48	625	0.22	0.35
AT2G06530	*vps2.1*	3675	elf18^{Ps}	+	44/66	1168	0.08	0.19
AT2G06530	*vps2.1*	3675	flg22^{Pa}	-	23/24	318	0.38	0.44
AT2G40930	*ubp5*	0	elf18^{Ps}	-	37/36	834	0.06	0.16
AT2G40930	*ubp5*	0	elf18^{Pv}	+	22/35	317	0.27	0.38
AT2G40930	*ubp5*	0	flg22^{Pa}	+	39/36	634	0.48	0.49
AT3G05200	*atl6*	-4646	elf18^{Ps}	+	16/43	344	1.00	0.67
AT3G06810	*ibr3*	0	elf18^{DC}	-	82/105	4632	0.37	0.43
AT3G06810	*ibr3*	0	elf18^{Pv}	-	57/94	2945	0.31	0.40
AT3G06810	*ibr3*	0	flg22^{Pa}	-	18/18	164	0.96	0.66
AT3G16030	*ces101*	5208	flg22^{Pa}	+	49/80	2183	0.28	0.39
AT3G25040	*erd2b*	-32	elf18^{Ps}	-	50/117	3128	0.48	0.49
AT3G25070	*rin4*	-8038	elf18^{Ps}	+	21/24	249	0.96	0.66
AT3G25070	*rin4*	-8038	flg22^{Pa}	-	16/35	379	**0.0447**	0.12
AT3G45290	*mlo3*	-170	elf18^{Ps}	+	18/35	308	0.90	0.65
AT3G45290	*mlo3*	-170	elf18^{Pv}	+	20/41	359	0.44	0.47
AT3G45290	*mlo3*	-170	flg22^{Pa}	+	22/40	415	0.72	0.59
AT3G45640	*mpk3*	-1043	elf18^{Pv}	-	17/27	273	0.30	0.40
AT3G46930	*at3g46930*	-1932	elf18^{Pv}	+	18/36	289	0.53	0.52
AT3G59830	*at3g59830*	11482	elf18^{DC}	+	18/18	164	0.96	0.66
AT3G59830	*at3g59830*	11482	elf18^{Ps}	+	32/53	591	**0.0200**	0.07
AT3G59830	*at3g59830*	11482	elf18^{Pv}	-	17/18	166	0.68	0.58
AT3G59830	*at3g59830*	11482	flg22^{Pa}	+	18/17	75	**0.0093**	**0.0395**
AT4G04450	*wrky42*	0	flg22^{Pv}	+	12/12	62	0.59	0.54
AT4G11900	*at4g11900*	0	elf18^{Pv}	+	37/41	682	0.45	0.47
AT4G19660	*npr4*	0	elf18^{Ps}	+	24/37	373	0.30	0.40
AT4G19660	*npr4*	0	flg22^{Pa}	-	8/9	40	0.74	0.60
AT4G21865	*at4g21865*	0	elf18^{DC}	-	24/12	161	0.58	0.54
AT4G21865	*at4g21865*	0	elf18^{Ps}	+	16/18	40	**0.0002**	**0.0024**
AT4G21865	*at4g21865*	0	flg22^{Pa}	+	18/21	96	**0.0081**	**0.0361**
AT4G21865	*at4g21865*	0	flg22^{PsHR+}	-	16/17	156	0.49	0.50

AT4G23160	*crk8*	0	elf18^{DC}	+	19/35	365	0.57	0.53
AT4G24240	*wrky7*	-12408	elf18^{Ps}	-	22/42	523	0.40	0.44
AT4G34150	*at4g34150*	0	elf18^{Ps}	-	22/48	605	0.34	0.42
AT4G38940	*at4g38940*	-9860	elf18^{Pv}	+	41/78	1548	0.78	0.61
AT4G40085	*at4g40085*	-12995	elf18^{Ps}	+	11/37	167	0.38	0.44
AT5G20480	*efr*	-785	elf18^{DC}	-	26/72	1859	**0.0000**	**0.0000**
AT5G20480	*efr*	-785	elf18^{Ps}	-	42/97	3342	**0.0000**	**0.0000**
AT5G20480	*efr*	-785	elf18^{Pv}	-	57/128	7255	**0.0000**	**0.0000**
AT5G20480	*efr*	-785	flg22^{Pa}	-	7/2	7	1.00	0.67
AT5G25910	*rlp52*	0	elf18^{Pv}	-	18/45	366	0.56	0.53
AT5G43380	*topp6*	0	elf18^{DC}	+	18/36	300	0.67	0.57
AT5G43380	*topp6*	0	elf18^{Ps}	-	6/6	20	0.82	0.62
AT5G43380	*topp6*	0	elf18^{Pv}	-	17/18	122	0.32	0.41
AT5G43380	*topp6*	0	flg22^{Pa}	+	60/92	2101	**0.0131**	**0.0497**
AT5G43900	*mya2*	0	elf18^{DC}	+	18/39	353	0.98	0.66
AT5G44510	*tao1*	-6300	elf18^{DC}	-	18/18	170	0.81	0.62
AT5G44510	*tao1*	-6300	elf18^{Ps}	-	55/98	3503	**0.0021**	**0.0131**
AT5G44510	*tao1*	-6300	elf18^{Pv}	-	18/18	192	0.35	0.43
AT5G44510	*tao1*	-6300	flg22^{Pa}	-	45/41	1295	**0.0011**	**0.0092**
AT5G44560	*vps2.2*	1458	elf18^{DC}	-	18/36	404	0.15	0.27
AT5G44560	*vps2.2*	1458	elf18^{Ps}	-	21/21	302	**0.0407**	0.11
AT5G44560	*vps2.2*	1458	elf18^{Pv}	+	85/81	3149	0.34	0.42
AT5G44560	*vps2.2*	1458	flg22^{Pa}	-	43/43	1054	0.27	0.38
AT5G45770	*rlp55*	1157	elf18^{Ps}	-	3/3	6	0.70	0.58
AT5G45770	*rlp55*	1157	flg22^{Pa}	-	33/54	988	0.40	0.45
AT5G45780	*at5g45780*	-734	flg22^{Pa}	-	35/54	1160	0.07	0.17
AT5G46220	*at5g46220*	0	elf18^{DC}	-	18/18	204	0.19	0.32
AT5G46220	*at5g46220*	0	elf18^{Ps}	-	29/23	466	**0.0141**	0.05
AT5G46220	*at5g46220*	0	elf18^{Pv}	-	36/56	1285	**0.0270**	0.09
AT5G46220	*at5g46220*	0	flg22^{Pa}	-	15/15	137	0.32	0.41
AT5G46270	*at5g46270*	74	flg22^{Pa}	+	16/32	259	0.96	0.66
AT5G46270	*at5g46270*	74	flg22^{PsHR-}	-	15/30	246	0.63	0.56
AT5G46450	*at5g46450*	-3492	flg22^{Pa}	-	18/35	327	0.83	0.63
AT5G46720	*aig2l*	11537	elf18^{DC}	-	21/21	163	0.15	0.28

AT5G46720	*aig2l*	11537	elf18^{Ps}	+	29/28	427	0.75	0.60
AT5G46720	*aig2l*	11537	elf18^{Pv}	+	18/21	233	0.22	0.35
AT5G46720	*aig2l*	11537	flg22^{Pa}	+	28/43	642	0.64	0.56
AT5G46720	*aig2l*	11537	flg22^{PsHR-}	+	21/21	226	0.90	0.64
AT5G47910	*rbohd*	2845	elf18^{DC}	-	18/18	259	**0.0016**	**0.0114**
AT5G47910	*rbohd*	2845	elf18^{Ps}	-	6/6	35	**0.0043**	**0.0228**
AT5G47910	*rbohd*	2845	elf18^{Pv}	-	29/30	576	**0.0324**	0.10
AT5G47910	*rbohd*	2845	flg22^{Pa}	-	49/64	2300	**0.0000**	**0.0000**
AT5G47910	*rbohd*	2845	flg22^{PsHR-}	+	12/12	70	0.93	0.65
AT5G52900	*makr6*	-3269	flg22^{PsHR-}	+	20/39	363	0.67	0.58
AT5G53010	*at5g53010*	0	elf18^{DC}	-	18/16	159	0.62	0.56
AT5G53010	*at5g53010*	0	elf18^{Pv}	-	75/82	3601	0.06	0.16
AT5G53010	*at5g53010*	0	flg22^{Pa}	+	18/17	146	0.83	0.63
AT5G53010	*at5g53010*	0	flg22^{PsHR-}	+	18/18	153	0.79	0.61
AT5G57330	*at5g57330*	2731	elf18^{DC}	-	16/36	317	0.58	0.54
AT5G57330	*at5g57330*	2731	elf18^{Pv}	+	74/136	4637	0.35	0.42
AT5G57330	*at5g57330*	2731	flg22^{Pa}	-	18/17	229	**0.0114**	**0.0454**
AT5G57340	*at5g57340*	-2266	elf18^{Ps}	-	36/51	979	0.60	0.55
AT5G57340	*at5g57340*	-2266	flg22^{Pa}	+	18/16	139	0.88	0.64
AT5G57345	*at5g57345*	-5587	elf18^{DC}	-	18/18	203	0.20	0.33
AT5G57345	*at5g57345*	-5587	elf18^{Ps}	+	18/36	177	**0.0064**	**0.0307**
AT5G57345	*at5g57345*	-5587	elf18^{Pv}	+	18/18	141	0.52	0.51
AT5G57345	*at5g57345*	-5587	flg22^{Pa}	+	12/12	36	**0.0387**	0.11
AT5G46330	*fls2*		flg22^{Pa}	-	34/36	0	**0.0000**	**0.0000**
AT5G46330	*fls2*		flg22^{PsHR-}	-	17/19	0	**0.0000**	**0.0000**
AT5G46330	*fls2*		flg22^{PsHR+}	-	3/3	0	0.10	0.21

Table S6
